# Draft Whole-Genome Sequence of *Serratia marcescens* Strain RM66262, Isolated from a Patient with a Urinary Tract Infection

**DOI:** 10.1128/genomeA.01423-15

**Published:** 2015-12-03

**Authors:** Roberto E. Bruna, Santiago Revale, Eleonora García Véscovi, Javier F. Mariscotti

**Affiliations:** a Instituto de Biología Molecular y Celular de Rosario (IBR-CONICET), Departamento de Microbiología, Facultad de Ciencias Bioquímicas y Farmacéuticas, Universidad Nacional de Rosario, Rosario, Argentina; b Instituto de Agrobiotecnología Rosario (INDEAR)-CONICET, Rosario, Argentina

## Abstract

*Serratia marcescens* strains are ubiquitous bacteria isolated from environmental niches and also constitute emergent nosocomial opportunistic pathogens. Here, we report on the draft genome sequence of *S. marcescens* strain RM66262, which was isolated from a patient with urinary tract infection in the Bacteriology Service of the Rosario National University, Rosario, Argentina.

## GENOME ANNOUNCEMENT

*Serratia marcescens* is an opportunistic human pathogen associated with urinary and respiratory tract as well as wound and eye infections, endocarditis, osteomyelitis, meningitis, and septicemia. Immunocompromised people and newborns are the most affected hosts. The incidence of *S. marcescens* infection has increased over the last years, mainly due to the acquisition of multiple antibiotic resistances (1–4). Despite its clinical prevalence, the factors and mechanisms that contribute to *Serratia* pathogenesis remain unclear. *S. marcescens* strain RM66262 is a nonpigmented clinical isolate from a patient with urinary tract infection from the Bacteriology Service of the Facultad de Ciencias Bioquímicas y Farmacéuticas of the Rosario National University, Rosario, Argentina (5).

In previous reports from our group, we demonstrated that this strain of *S. marcescens*, using an *in vitro* infection model of nonphagocytic cells, provokes an extracellular induction of autophagy mediated by ShlA pore-forming toxin, and then is able to internalize, survive, and replicate inside large membrane-bound compartments that recruit prototypical markers of autophagosomes (6–8). Earlier work from our group pointed to the *S. marcescens* Rcs system as a key player in the regulation of the expression of virulence determinants of the bacterium (5, 9). We also found that our strain RM66262 produces outer membrane vesicles (OMVs), which package and deliver a specific cargo of potential virulence determinants (10).

In this study, we present the draft genome sequence of *S. marcescens* strain RM66262. Genomic DNA was isolated from an overnight-grown liquid culture, using the Wizard genomic DNA purification kit (Promega). DNA was quantified using the Ultrospec 2000 spectrophotometer (Pharmacia Biotech). The genome sequence was obtained on an Illumina HiSeq 1500 instrument at INDEAR (Argentina), using a whole-genome shotgun strategy with 2 × 100-bp reads. Overall genome coverage of ~900-fold was obtained. The A5-miseq pipeline (11, 12) was used to perform read trimming and correction, contig assembly, crude scaffolding, misassembly correction, and final scaffolding.

The genome of *S. marcescens* strain RM66262 has 4,882,260 bp, with a total G+C content of 60.1%. Genome annotation was performed using the NCBI Prokaryotic Genome Automatic Annotation Pipeline (PGAAP) (13). The *S. marcescens* strain RM66262 genome has 4,520 genes; among the identified genes 4,366 are protein-coding sequence genes (CDSs), and 53 are pseudogenes. The genome also has 10 rRNA genes (5S, 16S, and 23S) and 79 tRNAs genes. The RAST server (14) predicted coding sequences belonging to 561 subsystems.

The information provided in the genome sequence of *S. marcescens* strain RM66262 will enable further studies to understand the mechanisms of pathogenesis, characterize virulence factors, and analyze gene expression regulation of this opportunistic human pathogen.

### Nucleotide sequence accession numbers.

This whole-genome shotgun project has been deposited at DDBJ/EMBL/GenBank under the accession number JWLO00000000. The version described in this paper is the first version, JWLO01000000, and consists of sequences JWLO01000001 to JWLO01000019.
